# Prognostic impact of the ratio of the main pulmonary artery to that of the aorta on chest computed tomography in patients with idiopathic pulmonary fibrosis

**DOI:** 10.1186/s12890-019-0843-5

**Published:** 2019-04-18

**Authors:** Ji Soo Choi, Sang Hoon Lee, Ah. Young Leem, Joo Han Song, Kyung Soo Chung, Ji Ye Jung, Young Ae Kang, Moo Suk Park, Young Sam Kim, Joon Chang, Song Yee Kim

**Affiliations:** Division of Pulmonology, Department of Internal Medicine, Severance Hospital, Yonsei University College of Medicine, 50 Yonsei-ro, Seodaemun-gu, Seoul, 03722 Republic of Korea

**Keywords:** Pulmonary artery, Idiopathic pulmonary fibrosis, Prognosis

## Abstract

**Background:**

In many clinical disorders, there is a relationship between the ratio of the diameter of the main pulmonary artery (mPA) to that of the aorta (Ao) on chest computed tomography (CT). The aim of this study was to determine if the mPA/Ao ratio at diagnosis is associated with the clinical characteristics and outcomes in patients with idiopathic pulmonary fibrosis (IPF).

**Methods:**

We retrospectively reviewed the diameters of the pulmonary artery and aorta on chest CT, clinical characteristics, and results of other examinations in 303 patients at the time of initial diagnosis of IPF at our tertiary care center between 2011 and 2015. The primary outcomes were death and lung transplantation. The patients were followed up until June 2017.

**Results:**

One hundred and eight patients (35.6%) died and 58 (19.1%) underwent lung transplantation during follow-up. The mean mPA and Ao diameters were 28.3 mm and 34.0 mm, respectively, and the mean mPA/Ao ratio was 0.84. Thirty-one patients (10.2%) had an mPA/Ao ratio > 1.0 and 182 (60.1%) had an mPA/Ao ratio > 0.8. Patients with an mPA/Ao ratio > 0.8 had a lower DLco value than those with an mPA/Ao ratio ≤ 0.8. In Kaplan-Meier analysis, patients with an mPA/Ao ratio > 1.0 or > 0.8 had worse outcomes than those with an mPA/Ao ratio ≤ 1.0 and ≤ 0.8, respectively.

**Conclusions:**

A higher mPA/Ao ratio based on 1.0 and 0.8 is associated with unfavorable prognosis in patients with IPF.

## Background

Idiopathic pulmonary fibrosis (IPF) is a chronic, progressive, and fatal disease. The natural course of IPF can be diverse, and prediction of the prognosis is difficult. Pulmonary hypertension (PH) is common in the advanced stages of IPF, and its presence is reported to be associated with decreased exercise capacity and high mortality in patients with IPF [1–3]. The definition of PH is based on data obtained by right heart catheterization [4], which is an invasive and costly procedure. Furthermore, transthoracic echocardiography is known to be a useful noninvasive method when screening for PH [5, 6] but is also a costly method and has limited accuracy for assessment of PH in patients with IPF [7, 8].

Measurement of the diameter of the main pulmonary artery (mPA) on CT of the chest has been reported to predict the presence of PH in patients with parenchymal lung disease, such as IPF or chronic obstructive pulmonary disease (COPD) [9–12], and the ratio of the diameter of the mPA to that of the aorta (Ao) > 1 has been reported to indicate a very high probability of PH [13]. Furthermore, in a previous study conducted in the US, an mPA/Ao ratio > 1 was associated with worse outcomes in patients with IPF, and measurement of the mPA/Ao ratio on chest CT assisted in evaluation of the risk and prognosis in these patients [14]. However, another study reported that the diameter of the mPA, that of the Ao, and the mPA/Ao ratio can vary according to ethnicity and lifestyle [15], implying that the mPA/Ao ratio may be different in Asian patients with IPF from that in their Western counterparts. The aim of this study was to determine if there is an association of the mPA/Ao ratio with clinical characteristics and outcomes in Korean patients with IPF.

## Methods

### Study design

We retrospectively identified 460 patients who were diagnosed or referred with IPF at our tertiary care university hospital in South Korea between January 2011 and December 2015. The diagnosis was confirmed clinically in 296 patients and pathologically in 164 patients by consensus among multidisciplinary specialists according to the 2011 American Thoracic Society/European Respiratory Society/Japanese Respiratory Society/Latin American Thoracic Association guidelines [2]. One hundred and fifty-seven patients who did not attend for follow-up after diagnosis were excluded, leaving data for 303 patients with IPF available for inclusion in the analysis.

The patients’ initial CT scans of the chest and medical records, including baseline characteristics, laboratory findings, 6-min walking distance, pulmonary function test (PFT) results at the time of diagnosis, and echocardiographic findings were reviewed to evaluate their associations with the mPA/Ao ratio. The primary outcomes were death and lung transplantation. The study protocol was approved by the institutional review board at Severance Hospital (IRB 4-2017-1219) and the need for informed consent was waived.

### Measurements

**Fig. 1:**
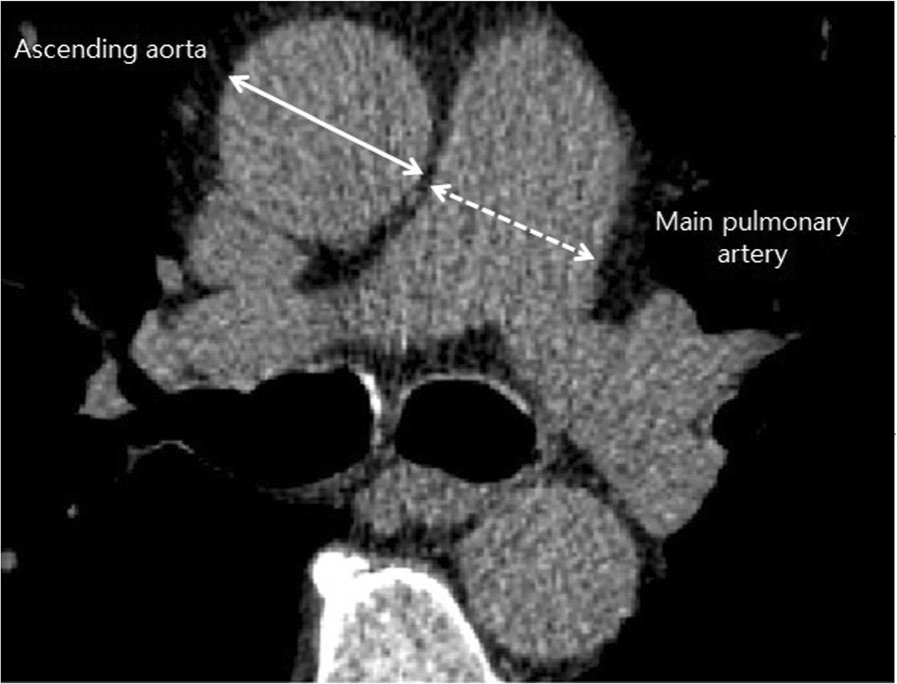
Measurement of the main pulmonary artery (mPA) and ascending aorta (Ao) diameters at the level of bifurcation of the pulmonary artery. The diameter of the mPA was defined as the widest diameter perpendicular to the long axis of the mPA and measurement of the ascending Ao was taken as the transverse diameter

CT scans of the chest were performed in all the patients at the time of initial diagnosis of IPF. The diameter of the mPA and that of the ascending Ao were measured at the level of bifurcation of the pulmonary artery. The diameter of the mPA was defined as the widest diameter perpendicular to the long axis of the mPA and measurement of the ascending Ao was taken as the transverse diameter (Fig. 1). Two reviewers who were blinded to the clinical data measured the mPA and Ao on the same CT image independently, and the average of two measurements was used to calculate the mPA/Ao ratio. The value of inter-observer correlation coefficient was 0.84 (95% confidence interval (CI), 0.80–0.87) for mPA, and 0.92 (95% CI, 0.90–0.94) for Ao.

We divided the patients into two groups according to whether they had an mPA/Ao ratio ≤ 1 or > 1 on the initial chest CT. An mPA/Ao ratio > 1 was considered to indicate the presence of PH [13]. The patients were then grouped according to whether the mPA/Ao ratio was ≤0.8 or > 0.8 because of a previous report indicating that Korean patients with COPD and an mPA/Ao ratio > 0.8 had clinical characteristics that differed from those in their counterparts with an mPA/Ao ratio ≤ 0.8 [16].

### Statistical analysis

The statistical analysis was performed using IBM SPSS version 23.0 software (IBM Corp., Armonk, NY, USA). Categorical variables are presented as the frequency and percentage and continuous variables as the median if the distribution was normal and as the interquartile range (IQR) if the distribution was non-normal. The GAP score, which includes sex, age, forced vital capacity (FVC) % predicted, and diffusing capacity of the lung for carbon dioxide (DL_CO_) % predicted [17] was calculated for each patient. The clinical characteristics of patients with a high mPA/Ao ratio and those with a low mPA/Ao ratio using cutoff values of 1 and 0.8 were compared by logistic regression analysis and the results are presented as the odds ratio and 95% confidence interval (CI). The primary outcomes were evaluated using Kaplan-Meier curve analysis. The association of the mPA/Ao ratio with the outcome was analyzed by multivariate analysis using a Cox proportional hazards model, and the results are presented as the hazard ratio (HR) and 95% CI. *P*-values ≤.05 were considered to be statistically significant.

## Results

### Baseline characteristics

Table 1 shows the baseline characteristics of the enrolled patients, who comprised 234 men (77.2%) and 69 women (22.8%) of mean age 67.2 ± 9.5 years. Two hundred and one patients (69.6%) were current or former smokers; these patients had a median 30-pack-year smoking history. The most common comorbidities were hypertension (33.0%) and diabetes mellitus (29.0%), followed by pulmonary tuberculosis (13.2%) and emphysema (12.9%). Less common diseases included ischemic heart disease (9.6%) and congestive heart failure (3.3%).

**Table 1:** Baseline characteristics of patients with idiopathic pulmonary fibrosis

Clinical characteristics	*n* = 303
Age, years	67.2 ± 9.5
Male sex	234 (77.2%)
BMI, kg/m^2^, median (IQR)	22.7 (20.1–24.9)
Ever-smoker	211 (69.6%)
Smoking, pack-years, median (IQR)	30.0 (20.0–42.0)
Underlying disease	
Hypertension	100 (33.0%)
Diabetes mellitus	88 (29.0%)
Pulmonary tuberculosis	43 (13.2%)
Emphysema	39 (12.9%)
Ischemic heart disease	29 (9.6%)
Congestive heart failure	10 (3.3%)
GAP score, median (IQR)	3.0 (2.0–4.0)
6MWD, m, median (IQR)	400.0 (308.5–480.0) (*N* = 152)
Pulmonary function at diagnosis	
FVC, %, median (IQR)	74.0 (59.0–86.0)
FEV_1_, %, median (IQR)	88.0 (71.0–100.0)
FEV_1_/FVC, %, median (IQR)	83.0 (77.8–89.0)
DL_CO_, %	69.0 (51.0–86.0)
Echocardiography	
Ejection fraction %, median (IQR)	66.0 (61.0–70.0) (*N* = 196)
Right ventricular pressure, mmHg, median (IQR)	28.0 (28.0–47.0) (*N* = 163)
Chest CT	
mPA diameter, mm, median (IQR)	27.9 (26.1–30.5)
Ascending Ao diameter, mm, median (IQR)	34.2 (31.6–36.7)
mPA/Ao ratio, median (IQR)	0.83 (0.77–0.90)

The data are presented as the number (percentage) unless otherwise indicated. 6MWD, 6-min walking distance

*Ao* aorta, *BMI* body mass index, *CT* computed tomography, *DL*_*CO*_ diffusing capacity of the lung for carbon dioxide, *FEV*_*1*_ forced expiratory volume in 1 s, *FVC* forced vital capacity, *IQR* interquartile range, *mPA* main pulmonary artery

The median GAP score was 3.0 (IQR 2.0–4.0) and the median 6-min walking distance was 400 m (IQR 308.5–480.0). The median FVC %, forced expiratory volume in 1 s (FEV_1_) %, and FEV_1_/FVC ratio were 74.0, 88.0, and 83.0, respectively. The median DLco % was 69.0 (IQR 51.0–86.0). The median mPA, Ao, and mPA/Ao ratio values were 27.9 mm (IQR 26.1–30.5), 34.2 mm (IQR 31.6–36.7), and 0.83 (IQR 0.77–0.90), respectively.

### Clinical characteristics according to mPA/Ao ratio

Table 2 shows the clinical characteristics of the patients according to whether the mPA/Ao ratio was ≤1 or > 1. Thirty-one patients (10.2%) had an mPA/Ao ratio > 1.0. The median mPA diameter was 27.6 mm in the group with an mPA/Ao ratio ≤ 1 and 32.9 mm in the group with an mPA/Ao ratio > 1. There was no difference in body mass index or smoking history between two groups. Right ventricular pressure and GAP score values were significantly higher in the group with an mPA/Ao ratio > 1 in univariate analysis but not in multivariate analysis.

**Table 2:** Clinical characteristics of patients with idiopathic pulmonary fibrosis and a main pulmonary artery to aorta diameter ratio ≤ 1 and > 1

Clinical factors	mPA/Ao ratio		Univariable analysis			Multivariable analysis		
	≤1 (*n* = 272)	> 1 (*n* = 31)	OR	95% CI	*P*	OR	95% CI	*P*
Age, years	67.9 ± 9.1	60.4 ± 10.0	0.92	0.89–0.96	< 0.001			
Sex, male	215 (79.0%)	19 (61.3%)	2.38	1.09–5.19	0.029			
BMI, kg/m^2^ (median, IQR)	22.7 (20.2–24.9)	22.5 (19.1–26.2)	1.01	0.91–1.12	0.848			
Ever-smoker	194 (71.9%)	17 (54.8%)	0.48	0.22–1.01	0.054			
Underlying disease								
Hypertension	94 (34.6%)	6 (19.4%)	0.45	0.18–1.15	0.095			
Diabetes mellitus	82 (30.1%)	6 (19.4%)	0.56	0.22–1.41	0.215			
Pulmonary tuberculosis	41 (15.1%)	2 (6.5%)	0.39	0.09–1.69	0.208			
Emphysema	37 (13.7%)	2 (6.5%)	0.44	0.10–1.91	0.270			
Ischemic heart disease	28 (10.3%)	1 (3.2%)	0.29	0.04–2.21	0.233			
Congestive heart failure	10 (3.7%)	0 (0%)						
GAP score	3.11 ± 1.24	3.75 ± 1.26	1.57	1.08–2.27	0.018	1.53	0.69–3.40	0.298
6 MW distance, m (median, IQR)	410.0 (322.5–480.0)	232.5 (95.0–555.8)	0.99	0.99–1.00.	0.032	0.99	0.99–1.00	0.190
PFT at diagnosis								
FVC, % predicted, (median, IQR)	75.0 (61.0–86.3)	55.0 (44.5–72.0)	0.95	0.93–0.98	< 0.001			
FEV_1_, % predicted, (median, IQR)	89.5 (73.0–100.0)	64.0 (51.5–81.0)	0.96	0.95–0.98	< 0.001	1.07	0.99–0.15	0.074
DL_CO_, %	70.9 ± 23.8	48.4 ± 23.1	0.96	0.94–0.98	< 0.001			
Echocardiography								
EF, %, (median, IQR)	65.0 (61.0–69.0)	67.0 (64.0–71.0)	1.08	1.01–1.14	0.022	1.09	0.93–1.27	0.285
RVP, mmHg, (median, IQR)	37.0 (27.8–46.0)	43.0 (33.0–52.5)	1.03	1.01–1.06	0.014	1.04	0.99–1.10	0.113
Chest CT								
mPA diameter, mm, (median, IQR)	27.6 (25.8–29.8)	32.9 (29.8–35.1)	1.38	1.23–1.54	< 0.001			
Ascending Ao diameter, mm, (median, IQR)	34.5 (32.0–36.9)	29.8 (27.7–32.8)	0.71	0.62–0.80	< 0.001			

The data are presented as the number (percentage) unless otherwise indicated. 6MWD, 6-min walking distance

*Ao* aorta, *BMI* body mass index, *CI* confidence interval, *DL*_*CO*_ diffusing capacity, *EF* ejection fraction, *FEV*_*1*_ forced expiratory volume in 1 s, *FVC* forced vital capacity, *IQR* interquartile range, *mPA* main pulmonary artery, *OR* odds ratio, *PFT* pulmonary function tests, *RVP* right ventricular pressure

Table 3 shows the clinical characteristics of the patients according to whether the mPA/Ao ratio was ≤0.8 or > 0.8. One hundred and eighty-two patients (60.1%) had an mPA/Ao ratio > 0.8. The median mPA diameter was 26.3 mm in the group with an mPA/Ao ratio ≤ 0.8 and 29.7 mm in the group with an mPA/Ao ratio > 0.8. In multivariate analysis, the DL_CO_ was lower in the group with an mPA/Ao ratio > 0.8 (odds ratio 0.97, 95% CI 0.95–0.99, *P* = .003). There was no significant difference in patient age, FVC, FEV_1_, or echocardiographic findings between the two groups.

**Table 3:** Clinical characteristics of patients with idiopathic pulmonary fibrosis and a main pulmonary artery to aorta diameter ratio ≤ 0.8 and > 0.8

Clinical factors	mPA/Ao ratio		Univariate analysis			Multivariate analysis		
	≤ 0.8 (*n* = 121)	> 0.8 (*n* = 182)	OR	95% CI	*P*	OR	95% CI	*P*
Age, years	68.7 ± 8.3	66.1 ± 10.0	0.97	0.95–1.00	0.019	0.96	0.91–1.01	0.136
Sex, male	102 (84.3%)	132 (72.5%)	2.03	1.13–3.66	0.018			
BMI, kg/m^2^, (median, IQR)	23.2 (20.8–26.6)	22.4 (19.8–24.8)	0.96	0.91–1.03	0.247			
Ever-smoker	91 (75.8%)	120 (66.3%)	0.63	0.37–1.05	0.078			
Underlying disease								
Hypertension	40 (33.1%)	60 (33.0%)	1.00	0.61–1.62	0.987			
Diabetes mellitus	30 (24.8%)	58 (31.9%)	1.42	0.85–2.38	0.185			
Pulmonary tuberculosis	15 (12.4%)	28 (15.4%)	1.29	0.66–2.52	0.466			
Emphysema	19 (15.7%)	20 (11.0%)	0.67	0.34–1.31	0.240			
Ischemic heart disease	8 (6.6%)	21 (11.5%)	1.84	0.79–4.31	0.158			
Congestive heart failure	2 (1.7%)	8 (4.4%)	2.74	0.57–13.1	0.208			
GAP score	3.07 ± 1.16	3.24 ± 1.31	1.12	0.91–1.37	0.280			
6 MW distance, m (median, IQR)	415.0 (356.3–477.5)	400.0 (246.3–480.0)	0.99	0.99–1.00	0.107			
PFT at diagnosis								
FVC, % predicted, (median, IQR)	77.0 (63.0–90.5)	71.0 (55.0–83.0)	0.98	0.96–0.99	< 0.001	1.01	0.97–1.05	0.735
FEV_1_, % predicted, (median, IQR)	94.0 (76.0–101.0)	83.0 (66.0–98.5)	0.98	0.97–0.99	< 0.001	1.01	0.97–1.04	0.805
DL_CO_, %	77.1 ± 23.6	62.7 ± 23.6	0.97	0.96–0.99	< 0.001	0.97	0.95–0.99	0.002
Echocardiography								
EF, %, (median, IQR)	64.0 (60.0–69.0)	66.0 (62.0–70.0)	1.02	0.99–1.05	0.294	1.16	0.93–1.45	0.187
RVP, mmHg, (median, IQR)	34.4 (25.0–43.0)	40.0 (31.5–48.0)	1.03	1.00–1.05	0.038	1.01	0.98–1.05	0.485
Chest CT								
mPA diameter, mm, (median, IQR)	26.3 (24.5–27.9)	29.7 (27.1–31.9)	1.49	1.34–1.65	< 0.001			
Ascending aorta diameter, mm, (median, IQR)	35.7 (33.9–37.7)	32.7 (30.4–35.0)	0.75	0.69–0.82	< 0.001			

The data are presented as the number (percentage) unless otherwise indicated. 6MWD, 6-min walking distance

*Ao* aorta, *CT* computed tomography, *DL*_*CO*_ diffusing capacity, *EF* ejection fraction, *FEV*_*1*_ forced expiratory volume in 1 s, *FVC* forced vital capacity, *IQR* interquartile range, *mPA* main pulmonary artery, *PFT* pulmonary function test, *RVP* right ventricular pressure

### Outcomes of IPF and associated risks

One hundred and eight patients (35.6%) died and 58 (19.1%) underwent lung transplantation during the follow-up period. The adjusted hazard ratios from the Cox proportional hazards analysis are shown in Table 4. A lower body mass index (HR 0.93; 95% CI 0.89–0.97; *P* < .001), a higher GAP score (HR 1.68; 95% CI 1.22–2.31; *P* = .002), and a higher mPA/Ao ratio (HR 3.06; 95% CI 1.85–5.07; *P* < .001) were significantly associated with a poor prognosis, including death and lung transplantation, in patients with IPF.

**Table 4:** Clinical characteristics associated with death or lung transplantation in patients with idiopathic pulmonary fibrosis (multivariate analysis)

Variable	Hazard ratio	95% CI	*P*-value
BMI, kg/m^2^	0.925	0.887–0.965	< 0.001
GAP score	1.676	1.218–2.306	0.002
mPA/Ao ratio	3.062	1.849–5.071	< 0.001

*Ao* aorta, *BMI* body mass index, *CI* confidence interval, *mPA* main pulmonary artery

**Fig. 2:**
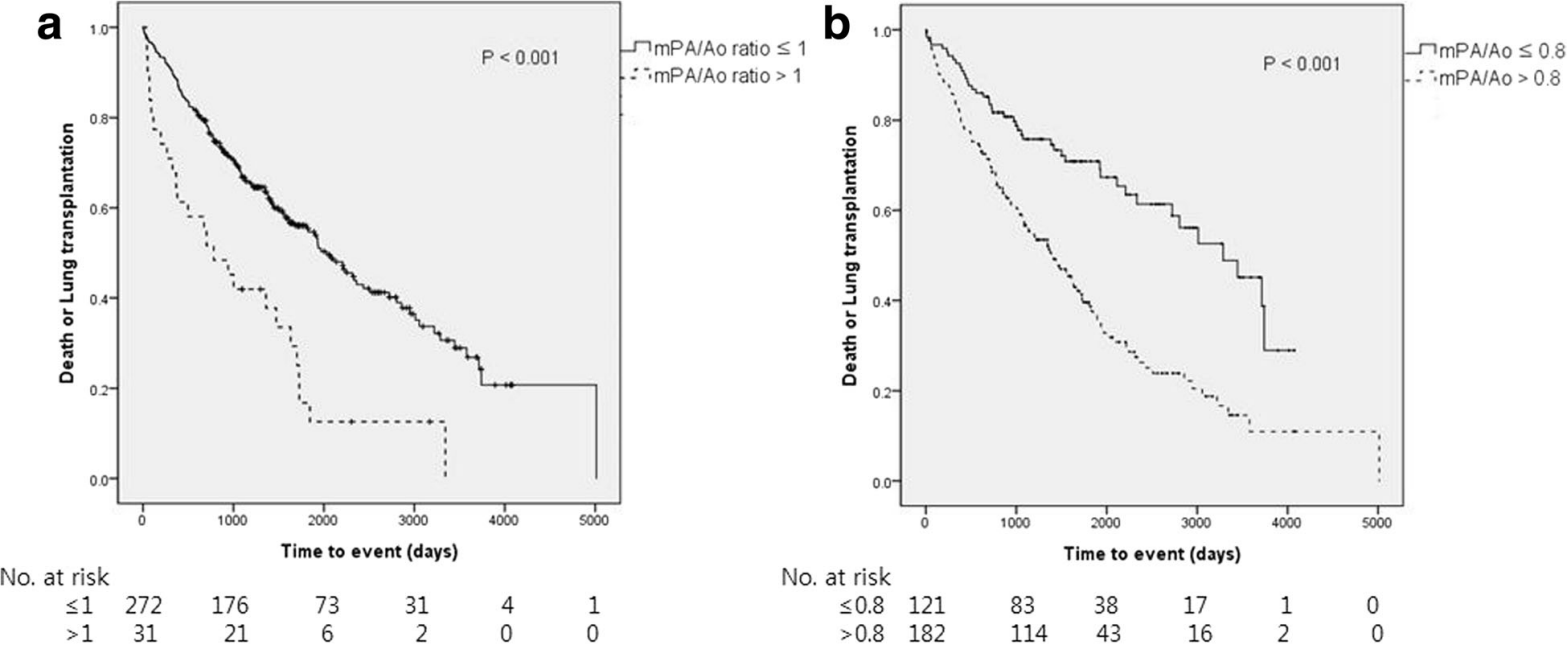
Kaplan-Meier analysis of time to death or lung transplantation in patients with idiopathic pulmonary fibrosis according to whether the ratio of the diameter of the main pulmonary artery to that of the aorta on chest computed tomography was **a** ≤1.0 or > 1 or **b** ≤0.8 or > 0.8 (*P* < .001)

Kaplan-Meier analysis of time to death or lung transplantation revealed that patients with an mPA/Ao ratio > 1.0 had a worse outcome than those with an mPA/Ao ratio ≤ 1.0. Furthermore, the patients with an mPA/Ao ratio > 0.8 had a worse outcome than those with an mPA/Ao ratio ≤ 0.8 (*P* = .003; Fig. 2).

## Discussion

The results of this study indicate that an mPA/Ao ratio > 1 is associated with worse outcomes, including death and lung transplantation, in patients with IPF. An mPA/Ao ratio > 0.8, which was the median value in patients with IPF, was also associated with a poor outcome.

IPF is a specific form of chronic and progressive fibrosing interstitial pneumonia of unknown cause, with a reported annual incidence of 0.8–76.4 per 100,000 population [18, 19] and presents as progressive aggravation of dyspnea and decreasing lung function. The prognosis for patients with IPF is poor, with a median survival of 2.5–3.5 years [2, 20, 21]. PH is common in patients with IPF and is also associated with an increased risk of death and a poor outcome [22–24]. In a previous study, the prevalence of PH in patients with IPF listed for lung transplantation was 32% by right heart catheterization, and there was a linear correlation between the mortality risk and mean pulmonary artery pressure [1].

In our study, univariate analysis revealed a relationship between a higher mPA/Ao ratio and a higher right ventricular pressure in patients with IPF. Although we could not perform right heart catheterization as part of our study, a higher right ventricular pressure is a known manifestation of PH [25, 26], so an association between the mPA/Ao ratio and PH is likely in patients with IPF. This finding is similar to those in previous studies in other pulmonary diseases [7, 9, 27, 28]. Iyer et al. reported that an mPA/Ao ratio > 1 on a CT scan in patients with COPD correlated with invasive measurements of pulmonary artery pressure and the presence of PH [29]. Schmidt et al. also reported that estimation of pulmonary artery diameter on chest CT could predict the presence of PH in patients with chronic pulmonary embolism [30].

We found a statistically difference in the GAP score between the group with an mPA/Ao ratio > 1.0 and the group with an mPA/Ao ratio ≤ 1.0 in univariate analysis. The components of the GAP score, i.e., sex, age, FVC % predicted, and DLco % predicted, have been validated as prognostic factors in patients with IPF [17, 31–33]. Furthermore, in our study, the mPA/Ao ratio was associated with a poor outcome, including death or lung transplantation, in patients with IPF. Overall, our findings suggest that the mPA/Ao ratio could be used to predict the prognosis of IPF and the associated risks, which is in agreement with previous reports. Shin et al. reported that an mPA/Ao ratio > 1 was associated with poor outcomes in Western patients with IPF and that calculation of the mPA/Ao ratio on chest CT might be used for risk stratification in these patients [14]. Similar results have been reported in patients with COPD. A high mPA/Ao ratio was associated with severe exacerbations of COPD [34] and was an independent predictor of mortality [35]. Given the results in patients with COPD, a high mPA/Ao ratio in patients with IPF could be related to acute exacerbation in addition to the possibility of PH.

We also investigated the prognostic value of the mPA/Ao ratio when based on a cutoff of 0.8. An mPA/Ao ratio > 0.8 was also associated with a worse outcome than an mPA/Ao ratio ≤ 0.8 in patients with IPF. One Korean study reported a mean mPA/Ao ratio of 0.87 and another reported an mPA/Ao ratio > 0.8 to be a risk factor for exacerbation of COPD [15, 16]. These results can be explained by differences in the body mass index, lifestyle factors, and/or the distribution of mPA/Ao values in the study populations. Therefore, a borderline mPA/Ao ratio in a Korean patient with IPF may have adverse clinical implications in terms of the prognosis. Our findings suggest that patients with IPF and an mPA/Ao ratio > 0.8 require close observation, including short-term follow-up evaluation with chest CT and PFT results as well as early consideration for lung transplantation in view of their poor prognosis.

This study had several limitations. The first is that it was conducted retrospectively at a single center. Furthermore, some data that might have influenced the results were missing, and several examinations, including transthoracic echocardiography and 6-min walking distance, were not performed in all patients. Second, there might have been some variation in the mPA and Ao measurements obtained on chest CT depending on the reviewer. However, we tried to overcome this limitation by having two reviewers measure the diameters of the mPA and Ao independently. Third, the proportion of patients who underwent transplantation was relatively high at our center, which could have introduced a degree of bias when evaluating the outcome. Fourth, we did not perform right heart catheterization, so it was not possible to correlate the hemodynamics with the mPA/Ao ratio on CT in detail. Nevertheless, our study also has some strengths, in that it shows that a high mPA/Ao ratio could be a prognostic factor in Asian patients with IPF and that a borderline mPA/Ao value could be clinically meaningful in these patients. Our findings may be relevant to patients when they are initially diagnosed with IPF, although prospective studies are needed for validation.

## Conclusions

We have identified that the mPA/Ao ratio on chest CT at the time of initial diagnosis of IPF has prognostic implications. An mPA/Ao ratio > 1 was associated with poor outcome in patients with IPF. In addition, an mPA/Ao ratio > 0.8 can be considered an early adverse prognostic factor in these patients. We suggest that the diameters of the mPA and Ao should be measured on chest CT at the time of initial diagnosis of IPF and that patients found to have a higher mPA/Ao ratio be kept under close observation.

## Abbreviations

Ao: Aorta
CI: Confidence interval
CT: Computed tomography
DL_CO_: Diffusing capacity of the lung for carbon dioxide
FEV_1_: Forced expiratory volume in 1 s
HR: Hazard ratio
IPF: Idiopathic pulmonary fibrosis
IQR: Interquartile range
mPA: Main pulmonary artery
PH: Pulmonary hypertension
